# Reflective Writing about Near-Peer Blogs: A Novel Method for Introducing the Medical Humanities in Premedical Education

**DOI:** 10.1007/s10912-021-09693-3

**Published:** 2021-04-19

**Authors:** Rachel Conrad Bracken, Ajay Major, Aleena Paul, Kirsten Ostherr

**Affiliations:** 1 Department of Family and Community Medicine, Northeast Ohio Medical University, 4209 St. Rte. 44, Rootstown, OH 44272 USA; 2 Section of Hematology/Oncology, University of Chicago, Chicago, IL USA; 3 Department of Medicine and Department of Pediatrics, Donald and Barbara Zucker School of Medicine At Hofstra/Northwell, Hempstead, NY USA; 4 Rice University, Houston, TX USA

**Keywords:** Narrative medicine, Premedical education, Near-peer learning, Reflective writing, Blogs

## Abstract

Narrative analysis, creative writing, and interactive reflective writing have been identified as valuable for professional identity formation and resilience among medical and premedical students alike. This study proposes that medical student blogs are novel pedagogical tools for fostering peer-to-peer learning in academic medicine and are currently underutilized as a near-peer resource for premedical students to learn about the medical profession. To evaluate the pedagogical utility of medical student blogs for introducing core themes in the medical humanities, the authors conducted qualitative analysis of one hundred seventy-six reflective essays by baccalaureate premedical students written in response to medical student-authored narrative blog posts. Using an iterative thematic approach, the authors identified common patterns in the reflective essays, distilled major themes, coded the essays, and conducted narrative analysis through close reading. Qualitative analysis identified three core themes (empathic conflict, bias in healthcare, and the humanity of medicine) and one overarching theme (near-peer affinities). The premedical students’ essays demonstrated significant self-reflection in response to near-peer works, discussed their perceptions of medical professionalism, and expressed concerns about their future progress through the medical education system. The essays consistently attributed the impact of the medical student narratives to the authors’ status as near-peers. The authors conclude that reading and engaging in reflective writing about near-peer blog posts encourages premedical students to develop an understanding of core concepts in the medical humanities and promotes their reflection on the profession of medicine. Thus, incorporating online blogs written by medical trainees as narrative works in medical humanities classrooms is a novel pedagogical method for fostering peer-to-peer learning in academic medicine.

## Introduction

The emergence of the mobile, social web in the early 2000s fundamentally changed how students learn, from K-12 through continuing medical education (Ruiz, Mintzer, and Leipzig 2006). A prominent feature of this new pedagogical landscape is the growth of digitally-connected peer networks for sharing resources and information. Recent research has shown the value of blogs and social media for fostering peer-to-peer learning in academic medicine (Lockspeiser et al. 2008; Cheston, Flickinger, and Chisolm 2013; Goldie 2016). As compared to teaching by senior clinical faculty, peer-to-peer and near-peer learning offer novel approaches to a range of objectives, including enhancing awareness of sociocultural context (Tamachi et al. 2018), improving clinical skills (Chou and Teherani 2017), increasing anatomical knowledge (Agius et al. 2018), and transitioning to clerkships (Knobloch et al. 2018). Within the traditionally hierarchical context of medical education, online peer communities can enable previously unheard voices, such as medical trainees, to gain expression (Eysenbach 2008). Further, research has demonstrated the value of studying online medical student communities on platforms such as WhatsApp to better understand the concerns of participants in real-time and in a social context (Coleman and O’Connor 2019).

Research on peer learning through online communities builds on literature in the field of narrative medicine. The practices of studying and creating narratives (Charon, Hermann, and Devlin 2016) have been identified as valuable methods for fostering some of the Core Competencies for Entering Medical Students established by the Association of American Medical Colleges (AAMC), such as empathy and ethical responsibility (Association of American Medical Colleges, “The Core competencies for Entering Medical Students”). Baruch (2013) has argued that creative writing should be seen as a medical instrument that provides “tools for clinical excellence and empathy” (459). Wald and colleagues (2019) argue that interactive reflective writing can help develop professional identity and resilience in medical students, while Lamothe and colleagues (2014) suggest that this practice may be protective against burnout. In addition to its role in medical education, Kanter (2008) has demonstrated that the practice of narrative medicine can be successfully used with students in the baccalaureate premedical setting, which offers more time and space to develop independent, creative thinking skills as well as strong moral reasoning and a commitment to social justice.

The availability of public blogs written by medical students as they reflect on their experiences in training provides a unique opportunity for premedical students to gain insight from near-peers about their intended profession and to hone the practice of reflective writing. Thus, extending foundational research in narrative medicine, this study hypothesized that medical student blogs present an underutilized opportunity for medical educators – and other students – to gain insights into the qualitative aspects of their experience. To test our hypothesis that near-peer narratives prompt valuable reflection on professional identity development and provide a novel means to introduce core medical humanities concepts to premedical undergraduates, we conducted a qualitative study that examined students’ responses to creative nonfiction narratives written by medical students for an online blog.

## Methods

This qualitative study was conducted in the context of an introductory, baccalaureate premedical humanities course. We assigned a set of readings that were originally written for an online, peer-reviewed blog run by and for medical students called *in-Training* (https://in-training.org/); these blog posts were later published as an edited collection by the same name (Major and Paul 2016). The authorship of the essays by near-peer medical students was not explicitly emphasized by the instructor. Students in the course were assigned seventeen short pieces (approximately five hundred words each) from *in-Training* alongside readings from widely used scholarly sources in the field of medical humanities, including anthologies edited by Cole et al. (2015) and Jones, Wear, and Friedman (2014), over the course of a fourteen-week semester (see Table 1 for a list of blog post titles). The readings were selected to supplement analytical essays assigned to address the five thematic units of the course: The History of Medicine and “Disease” vs. “Illness,” Narrative Medicine, Disability and Health Disparities, Science and Technology, and Religion and Spirituality.

**Table 1:** Readings assigned from the *in-Training* medical student blog

Author	*in-Training* Blog Post	Publication Date	URL	Total Number of Responses per Blog Post
Amladi A	“When a Patient’s Disease Strikes a Chord”	February 24, 2014	https://in-training.org/patients-disease-strikes-chord-4617	9
Ferguson B	“The Chair”	October 6, 2012	https://in-training.org/the-chair-245	13
Kanabur P	“Breaking Down the Barrier”	February 18, 2015	https://in-training.org/breaking-barrier-8293	31
Koti A	“Street Medicine”	April 21, 2015	https://in-training.org/street-medicine-8725	27
McDaniel L	“Clinical Culture Shock: Low Health Literacy as a Barrier to Effective Communication”	November 11, 2014	https://in-training.org/clinical-culture-shock-low-health-literacy-barrier-effective-communication-7788	26
Mouli M	“A Story of Love from Psychiatry”	March 20, 2014	https://in-training.org/story-psychiatry-5553	14
Niforatos J	“Fading Memories of Love and Martinis”	January 27, 2015	https://in-training.org/fading-memories-love-martinis-8180	13
Pham J. T. H	“A Night at the Homeless Shelter”	December 21, 2012	https://in-training.org/a-night-at-the-homeless-shelter-654	34
Pollard K	“Beta Amyloid Blues”	March 12, 2014	https://in-training.org/beta-amyloid-blues-5209	8
Salgado S	“Eyes: A Reflection from the First Month of Clerkships”	August 6, 2014	https://in-training.org/eyes-7477	15
Shier M	“Stars, Dollar Bills and Other Essentials”	October 9, 2014	https://in-training.org/stars-dollar-bills-essentials-7679	6
Shroff P	“The Inevitable”	September 29, 2013	https://in-training.org/the-inevitable-2634	11
Stifani B.M	“Diversity and Rhinos”	September 24, 2012	https://in-training.org/diversity-and-rhinos-211	43
Taylor K	“Why I Am in the Room”	January 23, 2015	https://in-training.org/room-8128	6
Tsai J	“Breeze”	February 17, 2015	https://in-training.org/breeze-8426	22
Tsai J	“A Lack of Care: Why Medical Students Should Focus on Ferguson”	December 2, 2014	https://in-training.org/lack-care-medical-students-focus-ferguson-8031	46
Yan J	“Exam Room 3”	January 8, 2014	https://in-training.org/exam-room-3-3754	19

Our dataset consisted of 176 reflective essays written by sixty-two premedical students over the course of four semesters (August 2016 to April 2018). Study participants consisted of zero first-year students, twelve (20%) sophomores, twenty-two (37%) juniors, and twenty-six (44%) seniors. The majority of students (n = 44, 73%) were Natural Sciences majors, followed by Social Sciences (n = 11, 18%), Humanities (n = 9, 15%), and Engineering (n = 3, 5%). The numbers of majors do not add up to 100% due to the participation of students pursuing double majors. Students were assigned to read seventeen *in-Training* blog posts, and then select three of them to explore through short, critical written reflections of approximately 250–500 words. The number of reflections written about each *in-Training* blog post is displayed in Table 1.

Our qualitative analysis was conducted in two stages. First, the research team identified which *in-Training* blog posts were selected by the premedical students for their reflective writing. Next, we sought to identify the predominant themes that emerged from the reflective essays. We began by reading through all of the essays to deductively identify a broad range of important themes. We identified approximately twenty to thirty initial themes and, on the basis of discussion, reached consensus about terminology that could be used to cluster and distill conceptually similar themes into three, comprehensive thematic categories: empathic conflict, bias in healthcare, and the humanity of medicine. We used these categories to code the dataset of 176 essays, with each essay coded by two reviewers. We collaboratively verified consistent application of codes through regular team meetings throughout our analysis. All of the coded essay segments were then subjected to narrative analysis by two members of the research team using close reading methods to explore the premedical students’ engagement with near-peer medical student writings, which revealed an additional, overarching theme: near-peer affinities.

## Results

Evidence of the pedagogical value of integrating near-peer narratives into a baccalaureate medical humanities course emerged from deductive, iterative analysis of our dataset. The premedical students focused their reflective writings on three major themes: empathic conflict, bias in healthcare, and the “humanity of medicine” (Pham 2012).^1^ In addition, a fourth, overarching theme of near-peer affinities appeared across all three of the major themes. We present the results of our narrative analysis of these themes below. Illustrative quotes from premedical student essays are referenced by the semester and year followed by the essay number (for example, F16-8 represents an essay written in the Fall semester of 2016, with the randomly assigned numerical identifier of 8). Selected quotations from student reflections are compiled in Table 2.

**Table 2:** Selected quotations from pre-medical student essays on the 3 major themes

Essay Identifier	Article	Reflective Essay Excerpt
**Empathic Conflict**		
F16-1	Tsai, “Breeze”	One story that caught my eye was “Breeze.” The summer before entering my senior year of high school, I participated in a research program at Bascom Palmer Eye Institute during which I had the opportunity to sit in on rounds one morning. An ophthalmologist presented his work in treating a patient from Nassau who had suffered horrendous eye trauma following a gas explosion. Slide to slide was transitioned through, each revealing a new photo of the gory reality of the injured man’s face. There was no mention of how the patient reacted to this accident, nor the worry his family must have felt, especially being in another country. I am ashamed to admit that I was drawn into the excitement of the technicalities of the surgical process, much like the rest of the sea of white coats, giving no thought to such implications. Only after reading that narrative do I recognize the dehumanization I absent-mindedly became enthralled in. The medical student says, “the idea that this career will desensitize me to grief and illness and death and dying terrifies me.” I, too, am terrified that without my noticing I will lose the ability to empathize and that grief will become “just a breeze.”
F16-2	Mouli, “A Story of Love from Psychiatry” Salgado, “Eyes: A Reflection from the First Month of Clerkships” Tsai, “Breeze”	Furthermore, multiple selections – such as those of Mouli, Salgado, and Tsai – comment on how much of a connection there should be with a patient in order to provide the best care possible. While it has become a popular opinion that becoming overly involved emotionally with a patient is as much of a problem as not being invested enough, I am of the belief that not being emotionally connected to a patient is far worse for the outcome of care than being too immersed. Simply speaking, if being an empathic physician truly resonates with the ideals of being a good physician, then worrying about being overly sensitive and emotional when it comes to the cases of certain patients should be far down the list of concerns when trying to care for that patient.
F16-4	Tsai, “Breeze”	I fear that I will be the one affected by the passing of a patient on a significant date in the patient’s life, while everyone around me is checking his or her cell phones without a care in the world. I know this disconnect is vital to maintain sanity as a medical professional, but for me, showing emotion is just as important.
F16-12	Yan, “Exam Room 3” Mouli, “A Story of Love from Psychiatry	I am not a person who likes to feel very much, I tend to push away negative feelings and emotions. I think it is part of how I push through the daily struggles, I drop all negativity from my life as soon as I can, and sometimes that involves suppressing my feelings. […] “Exam Room 3” hit me in a soft spot, since I have been dealing with a friend going through something similar, but “A Story of Love from Psychiatry” really made me feel things. Lately, I have been feeling rather alone, even though I have made many new friends and met amazing people, and the story of these people’s love was a little bit too much for me to handle dry-eyed.
F16-15	Tsai, “Breeze”	I was intrigued by the author’s advocacy of physician sensitivity in an era when medical professionalism and objectivity are celebrated over emotional attachment. This excerpt also gave me hope for the empathic and sensitive practice of medicine in the future because, like this author, I too value empathy for others as a vital quality in medicine and in life. I am beginning to see that the presence of emotion in the practice of medicine is a balancing act that, like any humanistic quality, can be simultaneously imperfect and precarious and beautiful.
F16-17	Tsai, “Breeze”	Over the summer, I had my first experience with death in a clinical setting. It hurt – it cut extremely deep. They were twin boys – both of healthy size. In what seemed like an instant, they were gone. They came into the world already asleep for eternity. My heart hurt for their mother who cried in agony, still unaware of her reality. I cried for their father who held their lifeless bodies in harrowing silence. Death is inevitable, but its suddenness hurts. Nonetheless, I find it both therapeutic and necessary to openly discuss death and dying in preparation for the journey ahead.
F16-32	Pham, “A Night at the Homeless Shelter” Koti, “Street Medicine”	These two narratives evidence that working within community settings and with marginalized populations can alter the attitudes of future healthcare providers. I think that the honest depiction of uncomfortable and judgmental emotions at the beginning of these stories legitimizes the poignant reflective moments at the end of each piece by tracing the transformative narrative arcs of students who are ultimately able to recognize humanity in the most uncelebrated of circumstances.
F17-7	Tsai, “A Lack of Care: Why Medical Students Should Focus on Ferguson”	Reading about Tsai’s response to the way death was treated in the hospital conference has shown me that I still have a long way to go before I can share such strong convictions about medicine and treating near-death patients. Furthermore, reading Tsai’s response has inspired me to want to show genuine care toward the well-being and the emotional state of my patients, as I agree with her in that physicians should not be desensitized to grief, death, and illness.
F17-13	Tsai, “Breeze”	Struggling with inexperience and the imposter syndrome is difficult enough, so adding on the fear of losing your empathy and humanity to others can be very stressful. To me these sentiments are very real, so reading first-hand accounts of people with similar fears and struggles is very interesting.
F17-19	Mouli, “A Story of Love from Psychiatry”	When I imagine a physician regarding a patient, and part of my draw to the profession, is intersecting with these people’s lives as their physician and being able to help them in some capacity, hopefully to relieve their suffering so they can live better again, all the while knowing that what they take into the room with me is a rich story of a life behind them, lived up until this point. They aren’t just a single, one-dimensional point in my life, in which I evaluate them just as they are; they are 3 dimensional even in my own timeline, and that’s something I want to always fully acknowledge. I believe that if everyone remembers that, it is easier to humanize and empathize with each other. As a physician, considering this about their patients is important.
F17-25	Salgado, “Eyes: A Reflection from the First Month of Clerkships”	While this passage is of a doctor recounting the eyes of his patients, this piece reminds me of the eyes of the doctors who treated my mother. Quite often before delivering yet another piece of heartbreaking news, I noted her doctors tense their body and look down or to the side for a brief moment if no other health practitioner was present. When another care provider was present in the room, I felt as if a doctor delivering news looked to the other nurse or doctor as if they were pleading. I would too if all I had to tell three young children was that the fate of their mother was up to divine intervention. I would plead to the other people in the room with my eyes to switch spots as the bearer of news while simultaneously pleading with them for any and all emotional support after seeing three pairs of young eyes look back with the maturity that the fact that their mother’s death was inevitable had been internalized for too long.
F17-31	Tsai, “Breeze”	Jennifer’s internal struggle here is something that I’ve thought about a lot ever since I decided to become a pre-med. At first, I used to wonder how doctors can maintain such a stoic outlook in the face of some clearly emotional decisions. What is the point of a brusque doctor? Shouldn’t we show some empathy and compassion to the patient who’s suffering? Why should treatment only be about the biological symptoms? I thought there was genuinely something wrong about doctors who failed to show empathy and compassion to their patients. It was detrimental. However, when I think about it now, I can kind of see why doctors may attempt to put some objectivity and emotional distance between themselves and the patient. Doctors are human beings. If you had to face illness and death regularly and if you were continually emotionally affected by it, doing your job as a physician would become very difficult. Keeping that emotional distance is not a weakness; it’s almost a necessity that allows a physician to work to the best of his or her ability. If I was in Jennifer’s place, I would probably also be shocked by the general attitude of the presenter and audience, but this desensitization, which can also be seen as a type of professionalism, can only come with experience that a first year medical student does not have. There is value in grief; I don’t believe that it should be disregarded completely. However, at the same time, there is a purpose behind the way doctors act around their patients. If that method ultimately helps the doctor perform his or her duty as effectively as possible, then I would still say it’s a job well done.
F17-40	Ferguson, “The Chair”	In fact, encouraging emotions other than that of despair, sadness, confusion, or confliction that are typically associated with hospitalization allows for there to be a balance to the detached and professional attitudes physicians are trained to convey. Thus, a positive atmosphere benefits patients and physicians, effectively reviving the humanistic aspect of patient care that calls for attention to the emotional needs of a person. I find this particularly important because in a day and age when check-ups are rushed, ‘relationships,’ if they can even be deemed as such, with physicians become increasingly superficial, and the lack of genuine interactions lead to many modern issues such as a lack of trust, misunderstanding, and an overall lack of effective patient treatment.
S17-1	Mouli, “A Story of Love from Psychiatry”	The medical students who reflected on these experiences, however, found it difficult to let go of the emotions behind each patient’s unique story. They were frustrated by how easily physicians diagnosed them and referred them to someone else, and simply moved on to their next task or the next patient file. Death seems to be a recurring theme and although these medical students often go through emotional discomfort upon witnessing death, they eventually come to terms with it.
S17-2	Shroff, “The Inevitable” Tsai, “Breeze”	The medical students learned to hear and understand their patients more than just examine them. They learned to be communicative and open-minded. Moreover, they learned that it was okay to be emotional and to feel.
S17-3	Taylor, “Why I Am in the Room”	When this reading mentions how Mrs. B “growls” and “tries to bite,” it brings up animalistic traits. This implies how health professionals no longer perceive this demented patient as fully human and may unintentionally or intentionally dehumanize the patient through treatment of the patient.
S17-4	Tsai, “Breeze”	This selection was somewhat distressing to read due to the vast majority of readings that focused on death. The fact that I felt emotionally involved however, is testament to the value of narratives. Narratives from medical students are particularly relevant to us pre-meds, because we can easily imagine ourselves in their shoes a few years in the future. The naivety of these stories sometimes struck me – many pre-meds aspire to save lives and cure illnesses, and yet the reality often strays far from that ideal. The readings prompted me to reflect on my own clinical experience with death. On a hospital rotation, I once saw the body of a young man who had just died in the shock room from a drive-by shooting, a gunshot wound to the neck. Observing as they took pictures of the wound, I felt numb then and much less distraught than expected. It wasn’t until I happened to see his family members sitting together on the couch through the window of the consultation room that I truly realized it: he was dead. My stomach dropped then, from the split-second glimpse of the haunted look in their faces. Eyes hot, I immediately averted my gaze like I’d witnessed something forbidden. If I hadn’t, I would’ve cried.
S17-8	Niforatos, “Fading Memories of Love and Martinis”	When my great-grandmother suffered from dementia, I would engage in long thoughts about what happened in her mind, the importance of my own memories, and so much more. When the medical student began to explain how everyone is always aware of mortality, I completely understood. I enjoyed his story and all others. I often used to think that doctors shouldn’t have any feelings or emotions for their patients in order to not become emotionally attached, so as a pre-medicine student who worried about this, all of the stories reassured me that these medical students do feel something for their patients.
S17-10	Ferguson, “The Chair”	We technologize and over-work doctors as much as patients are dehumanized in the face of a further technologizing country and we really do forget that doctors are human and that they must form a connection with their patients, and then in many cases lose them. I feel nothing short of ignorant for forgetting to consider them in the applications of narratives to medicine. By making the patient the owner of the narrative you ignore the feelings and needs of the human treating them which is just as much a part of medicine and treatment as the patient is. You can watch what a doctor goes through as a patient and as an audience and as a potential medical student and almost never think of the emotional impact or adjustments that have had to be made because of working within medicine.
S17-22	Koti, “Street Medicine” Pham, “A night at the homeless shelter”	I have always been interested in people’s stories, especially people who are often ignored. People often forget that homeless people have a life; they have hopes, dreams, and fears. As a physician, I would want to serve people who need my services the most, and listen to their stories. I read a book called “Listening is an act of love” and the book was a series of interviews with everyday people. This book had a big impact in my life because for the first time I was able to hear the stories of everyday people. They were not big heroes, or industry captains, or members of the elite; they were ordinary people with funny, uplifting, and tragic memories and experiences. Thus, the stories by Pham and Koti resonated with me because they also tried to help the poorest in our society while listening to their stories. Racial, economic, and social justice begin with the individual experience; they all originate in the suffering of an individual because of a societal barrier. In order to make progress in these realms, we must first listen to the stories of the individual.
S18-2	Tsai, “Breeze”	On one hand, the narrator worries about developing a lack of empathy and having that affect one’s character or ability to experience grief; on the other hand, the narrator mentions the idea of being consumed by empathy, which then affects the physician’s emotional state and self-care. This prompts us to think about emotional distance from a patient as a defense mechanism.
S18-6	Tsai, “Breeze” Shroff, “The Inevitable” Shier, “Stars, Dollar Bills, and Other Essentials”	“Breeze” stirred up a sentiment that I worry about constantly. Even in EMS, I’ve become desensitized to accidents, blood, and rather gross-looking things. I’m afraid that I’ll forget to think about and respect the weight of a death if I come in contact with it too often. Jennifer thinks about the fact that the mother died on her daughter’s wedding day and the baggage that gives the bride and her husband (Tsai 2015: 293). I didn’t even think to consider that until I read it. This makes me fear for what will happen when I become a physician, but I’m glad I’m learning to think about this now. Many of the other [readings] made me think about mortality: what it means for me, and how I would respond to the death of someone I knew or loved. These readings made me think back to my own experience with patient death when I was volunteering at a hospice. I don’t think the death hit me as hard because I wasn’t there for the moment, nor had I gotten to know the patient very well yet, but it still made me think about how I will react in the future when more patients die under my care.
S18-7	Tsai, “Breeze”	I was shocked to read about the desensitization among physicians, and it’s hard to grasp that one day, the idea of death – arguably the most fearful thing of life – will no longer phase me. As Tsai says, I hope that these experiences never become a mere breeze in my existence.
S18-8	Yan, “Exam Room 3”	I think these readings did a great job of portraying the emotional conflicts many young medical students have and their question about mortality. For someone who is not a doctor, it makes it easier to understand why some doctors act the way that they do, it is not because they are cold and unfeeling, but because they are trying to protect and preserve their own emotional state.
S18-13	Jennifer Tsai, “Breeze”	Physicians must walk the fine line between being emotional and being empathetic/sensitive. It is a disservice to their humanity and to their patients for them to become desensitized completely – it dehumanizes both parties and undermines the very reasons many physicians entered this field. Physicians must learn to balance emotion and sensitivity, to care about patients and treat them with respect and dignity rather than a case file, but at the same time, not letting their lives being defined by their patients. Medicine needs to stay human – the physicians are human, the patients are human – neither are machines, both have emotions, and each deserves dignity and respect.
S18-38	McDaniel, “Clinical Culture Shock: Low Health Literacy as a Barrier to Effective Communication”	McDaniel's narrative reflects some thoughts that I’ve had myself just from volunteering. Her quote “we can’t always help people in the grand ways we once pictured” (McDaniel 2014) resonated with me a lot. During my time at the hospice, there were often times patients who had stories to tell of their past or of how they don’t want to be there. However, there’s nothing I can really do other than listen, or perhaps try to distract them with stories of my own. There were definitely people I couldn’t help in the way I wanted to.
**Bias**		
F16-18	Tsai, “A Lack of Care: Why Medical Students Should Focus on Ferguson”	The idea of systematic racism in “A Lack of Care: Why Medical Students Should Focus on Ferguson” brought me to contemplate the creation and spread of stereotypes, and how minority groups try to escape those ideas set by society. Growing up in Miami, I have never really experienced any kind of discrimination for being Hispanic. However, I did see the influence of racism within my own family. Every time I drove through a predominantly African American area, my mom would click the lock button of the car several times. When a black man parked next to us, my grandmother instructed me not to get out of the car until he was gone. I have been told by my family not to date an African American. Amongst many of the Hispanic people I know, there is even a gesture for signaling when someone is black (rubbing the index finger of one hand against the forearm of the opposite arm). As a child, I unconsciously took part in this blatant racism. When I noticed that my darker-skinned nanny ate a lot of M&Ms, I refused to eat the candy which I once loved for fear of turning black. Now that I have grown, I have come to recognize this issue and have made strides to call out such acts. I ask my mom to think about why she locks the car multiple times and why she feels unsafe. I got out of the car against my grandmother's will to show her that her racist perception is unfounded. I refuse to acknowledge demeaning gestures. However, I cannot help but wonder whether this racism towards African Americans by my Hispanic family is a way to deflect their own discrimination – a way for the Cuban minority to establish dominance over another group. This is not justification, but rather a theory as to why the issue of systematic racism is so inherent to society.
F16-21	Tsai, “A Lack of Care: Why Medical Students Should Focus on Ferguson”	The events in Ferguson were a huge eye-opener for me about the state of the nation that I live in, especially as a young African American female. It was disappointing to say the least and extremely frustrating. Combining events such as these with the seemingly obvious gaps in healthcare for my race is enough to make me ashamed of this country.
F16-24	Tsai, “A Lack of Care: Why Medical Students Should Focus on Ferguson”	[M]edicine is often viewed as above discrimination or immune to racism – we view doctors as scientists, rational and highly educated, instead of as people with backgrounds and upbringings that have exposed them to the same harmful stereotypes as everyone else.
F16-27	Tsai, “A Lack of Care: Why Medical Students Should Focus on Ferguson”	[M]edicine and health care [are] political issue[s] because all institutions such as hospitals and medical schools are vulnerable to systemic racism.
F16-30	Koti, “Street Medicine”	Over spring break, I went to Washington D.C. on an Alternative Spring Break Program with a group of students from Rice. On one of the days, we were with a physician who performed the same tasks as this medical student was performing. We walked around the entire city and stopped by every homeless individual we saw. We gave them a meal and as the doctor attended to their medical needs, we talked to them about their life and what brought them to where they were today. These individuals were so kind and open and shared their stories with us eagerly. I felt as if they were longing to have someone listen, to treat them as more than society saw, to treat them as human beings. I realized, through this experience, my implicit bias towards homeless individuals and how unjust I was in my thoughts.
F16-33	Tsai, “A Lack of Care: Why Medical Students Should Focus on Ferguson”	Many people seem to think that medicine exists in a vacuum, that medical education is scientific and therefore unbiased. However, even when you account for the fact that science is as subject to bias as any field, medicine is more human still because it focuses on the interaction between doctors and patients. Tests and technology are equalizers, but the primary mode of diagnosis still revolves around doctors listening to what patients are saying.
F16-34	Tsai, “A Lack of Care: Why Medical Students Should Focus on Ferguson”	I have recently found myself very much concerned and dismayed about the dehumanization and blatant disrespect of the black body. […] I always think that on any given day, one of those hashtags could belong to my father, my aunt, my brothers, my cousins, my boyfriend – to any of the men that I love so dearly.
F16-41	McDaniel, “Clinical Culture Shock: Low Health Literacy as a Barrier to Effective Communication”	Larger structural changes can be made to better accommodate the diversity of patients and individual providers can do much to make these patients feel more included.
F17-4	Tsai, “A Lack of Care: Why Medical Students Should Focus on Ferguson”	[T]he real issue isn’t the individuals who stick out for committing crimes of racism, but the system that made their implicit biases. This is a really important turning point in my own thoughts about this issue. […] [H]armful biases happen in everyday situations and though they aren’t as obvious, they matter and lead to statistically significant outcomes.
F17-11	Koti, “Street Medicine”	The image of the storm displacing homeless populations in Tampa is something that really resonated given the impacts of the recent hurricanes in Houston and even in Florida and Puerto Rico. The week after Harvey, I was leaving the bus stop in downtown Houston to come back to my dorm. Usually there are a few homeless people under the small overpass, but this time it was flooded on the sides. Instead a large group of people had gathered around the bus station and were sitting around there. The station employees were outside the door making sure they did not come too close. Frankly, I was scared. It was past midnight and I was waiting by myself as people were approaching me asking me for money. I just wanted to get out of there. Reading this story really made me reflect on my attitude toward those homeless people that night. I had no consideration for the fact that they had been displaced from their homes by the storm. In addition, these people who are already less fortunate than most are offered little aid. Instead, they are further oppressed as the system works against them and limits their opportunities for social mobility. This story was really short but it really inspired me and reminded me of the importance of helping others, especially those less fortunate than yourself.
F17-14	Tsai, “A Lack of Care: Why Medical Students Should Focus on Ferguson”	Changing a system that is rigged towards specific groups can pose a real challenge, which is why I think reading pieces like this one is important. Being aware of society’s innate racism, as well as the implicit biases that we carry, is the first step towards changing a system that is always providing disadvantages to certain people based on their group membership.
F17-20	Tsai, “A Lack of Care: Why Medical Students Should Focus on Ferguson”	The reality is that systemic racism is rampant in society, but it does not just affect political systems. These biases also exist within the health care system and physicians, subtly and not so subtly shaping their quality of care and the access to care for people of color […] Medical students should be educated on the social context of medicine, and be aware of the many biases that could seep into their practice.
F17-27	Tsai, “A Lack of Care: Why Medical Students Should Focus on Ferguson”	The issue of systemic racism is so ingrained into the lifestyle and beliefs of all [of us] and it scares me that doctors are not immune.
F17-32	Pham, “A Night at the Homeless Shelter”	Reading these selections has made me more aware of my own implicit biases. […] I’m grateful to be reading so much about implicit biases, because it has made me acutely aware of my own biases and how it may affect the kind of doctor I become.
F17-33	Stifani, “Diversity, and Rhinos”	I never realized how limiting western medicine could be. We question cultural competency in physicians and in our relationships, but I don’t think we ever really talk about how medicine as an institution tends to favor western thought. […] [I]t’s not your own personal bias that is coming into play. It’s a supposedly objective test that’s creating conclusions based on bias perpetuated by western thought in medicine. […] Implicit bias is institutionalized in medicine through these tests. […] It’s important to understand the limitations of western medicine when it comes to treating diverse patients.
F17-35	Tsai, “A Lack of Care: Why Medical Students Should Focus on Ferguson”	As a black woman, these readings make me extremely angry and resentful of a system that I and many other minority students are working hard academically to ultimately become a part of.
S17-13	Tsai, “A Lack of Care: Why Medical Students Should Focus on Ferguson” Pham, “A Night at the Homeless Shelter” Koti, “Street Medicine”	Mentally, all the patients become representative of a certain group and each case becomes mundane for the physician. Physically, detecting the nuances of each case that contributes to the patient’s complications can become very difficult. Emotionally, the patient satisfaction can be low, especially with the detachment that the patient and physician probably experience between each other. I can easily [see] many other negative effects of physicians acting on their predispositions instead of critically thinking and recognizing their bias. Personally, racism and other social justice issues have become a large part of my life in terms of extracurricular activities and more generally, what I do with my time. Although I saw a vague connection between this passion of mine and medicine, seeing the connection blatantly made for me through these texts made it so much more real. I realize that I can combine my interests in social justice with medicine quite easily and effortlessly whether it be through advocacy, service, or more.
S17-14	Tsai, “A Lack of Care: Why Medical Students Should Focus on Ferguson”	It’s frustrating to me that physicians do not take the time out of their day to check their biases and think about how that translates to poor care. […] If you truly cared about your patients and doing what is best for them, and if you understood medicine to be as social as it is biomedical, wouldn’t racial profiling be something of large importance to watch for?
S17-23	Koti, “Street Medicine”	As a student now quite dedicated to the mission of public health, I am always shocked at how elitism and classism function so vigorously within medicine and health care. As though only people that can afford to pay for health insurance or procedures out of pocket deserve medical attention.
S17-25	Tsai, “A Lack of Care: Why Medical Students Should Focus on Ferguson”	The fact that physicians constantly marginalize certain races (whether they are aware of it or not) when practicing motivates me to become more conscious of these acts when I go into the professional field.
S17-32	Stifani, “Diversity, and Rhinos” McDaniel, “Clinical Culture Shock: Low Health Literacy as a Barrier to Effective Communication”	While standardized tests or jargon may be used to communicate complicated medical issues, biomedicine has not reached the point where it meets the patient in the state that they are in. The diverse perspective of the patient and the experiences that they have had dictate the manner in which health care ought to persist, whether that means through language, explicatory jargon, or method of questioning. Since health care is dependent upon preserving [an] individual’s ability to live, inequitable delivery of care creates a system that excludes those that do not fit into the prototypical mold of Westernized medicine.
S18-18	Tsai, “A Lack of Care: Why Medical Students Should Focus on Ferguson”	The statistics that [Jennifer Tsai] shared really surprised me and I wondered if doctors were really this racist and rooted in their outdated social values and beliefs. However, Tsai later […] speaks about how even those who are anti-racist may be subject to experiencing implicit biases and I felt that this could explain for a lot of the discrepancies in care.
S18-19	Tsai, “A Lack of Care: Why Medical Students Should Focus on Ferguson”	Medical education needs to handle this topic [racial bias] by addressing not just the biomedicine in its curriculum, but also the social context of medicine.
S18-22	Tsai, “A Lack of Care: Why Medical Students Should Focus on Ferguson”	[W]e automatically assume that the health care system is more ‘just’ because it is based on a rational and objective science. This is far from the truth.
S18-29	Tsai, “A Lack of Care: Why Medical Students Should Focus on Ferguson”	Although medicine may be seen as an objective field, rooted in science and biomedicine, health care is not, and needs to be recognized and addressed in social contexts – taking into account socioeconomic disparities, marginalized populations, and biases that permeate society. I never thought about how [the biases that permeate society] may affect health care, and how widespread this bias is truly scares me. It extends to me.
**Humanity of Medicine**		
F16-18	Tsai, “A Lack of Care: Why Medical Students Should Focus on Ferguson”	The idea that medicine is “race-blind” is challenged, showing how societal perception influences physicians whether consciously or unconsciously. The need for a more holistic medical education which recognizes and addresses these issues is brought up.
F16-19	Tsai, “A Lack of Care: Why Medical Students Should Focus on Ferguson”	Ultimately, doctoring is an inherently social discipline that revolves around patient-doctor interaction; pretending medicine is a rational exercise denies its role as a social force and allows its continued participation in structural racism to go undiagnosed.
F16-31	Koti, “Street Medicine” Tsai, “A Lack of Care: Why Medical Students Should Focus on Ferguson”	Koti’s piece also shows how homeless people have multifaceted lives, a fact that healthcare practitioners often forget in their laser focus on diagnosing medical problems; his conversations with his patients about relationship issues help the reader better understand how homelessness is one of many identities a person can hold. […] [M]edical schools must make a greater effort to educate their students in understanding the patient as a whole, including their sociopolitical environment and their cultural norms, so that physicians in the real world are equipped to deal with a diverse patient population and can fight structural bias. […] Medicine is thought of as a sociopolitical vacuum because physicians are taught to treat everyone with the same amount of compassion and empathy; however, medicine exists among all other cultural and political factors, including implicit biases and stereotypes from the social world. Therefore, it is important to acknowledge and actively work to demolish these stereotypes within medicine and give all patients the care and treatment they need.
F16-33	Tsai, “A Lack of Care: Why Medical Students Should Focus on Ferguson”	When I volunteered in an emergency room, I witnessed the casual way in which patients who complained of pain were dismissed. I did not have access to medical records, but one patient I particularly remember was an older black lady who was crying because she was in pain. She said nobody had performed an examination on her, and when I asked a nurse, she waved me off, saying, “You get an eye for figuring out who’s faking it for drugs.” I was alarmed by this, but assumed she was correct, as I had almost no power in the situation. Whether or not this old woman was addicted to painkillers, wasn’t it the duty of medical professionals to trust her and give her the benefit of the doubt? When filtering through the hundreds of patients who are turned away for being fakers, isn’t it possible that a couple of them were telling the truth?
F16-35	Stifani, “Diversity, and Rhinos”	I think that even if a patient can’t understand you (the physician), the act of looking at them when you speak and talking to them directly can help create a connection. Facial gestures, eye contact, and other nonverbal signs are just as important in communication as the language itself and should not be undervalued.
F16-49	Stifani, “Diversity, and Rhinos” McDaniel, “Clinical Culture Shock: Low Health Literacy as a Barrier to Effective Communication”	While these students initially exhibit a desire to achieve an ideal diagnosis and treatment through effective communication with patients, they discover that personal interaction with patients requires a type of communication that engages patients on a level that transcends a biomedical understanding of the patient’s symptoms and history. […] The student who grapples with the impediment of low health literacy to a patient’s satisfaction and understanding of his healthcare also realizes that physicians must be able to understand the way that their patients communicate in order to shape their explanations and questions into interactions that satisfy patients’ expectations and make them comfortable.
F17-21	Kanabur, “Breaking Down the Barrier”	Patients may have a very different perception of their condition and it usually doesn’t involve data and numbers and labs. This perception is important because it is what dictates how they deal with it and live their lives with it, and so the doctor needs to know this story, which they can get only from asking the why questions.
S17-30	Stifani, “Diversity, and Rhinos” McDaniel, “Clinical Culture Shock: Low Health Literacy as a Barrier to Effective Communication”	Instead of language, linguistics and culture is the reason why there are issues with communication. As doctors, we must understand that communication is not just about speaking an understandable language, but is also about knowing how to converse interculturally. Mannerisms and the basic knowledge base of people from different cultures are vastly different, and this is one of the main reasons why doctors cannot just study the biology of disease, but also the sociology of it as well. It is obviously impossible to be able to communicate with all cultures, but doctors must know what resources they can use in order to bridge this gap in order to provide the best treatment.
S18-29	Tsai, “A Lack of Care: Why Medical Students Should Focus on Ferguson”	Although medicine may be seen as an objective field, rooted in science and biomedicine, health care is not, and needs to be recognized and addressed in social contexts – taking into account socioeconomic disparities, marginalized populations, and biases that permeate society. It needs to become something we train all health care professionals to be aware of, only then can we strive for equal and fair treatment.
S18-32	Pham, “A Night at the Homeless Shelter”	“A Night at the Homeless Shelter” brought up the idea of listening to the patient’s full story, which connects to the idea that we discussed during the first portion of this class about listening to a patient’s narrative rather than just isolating the disease. Working with a population so different from oneself can be an intimidating concept. However, listening to what a person who may come from a different background than ourselves can provide a greater understanding of who they are and make interacting with them seem less daunting.
S18-39	Stifani, “Diversity, and Rhinos”	We often associate “cultural competency” with language proficiency. For example, it’s often encouraged for pre-med students to study Spanish or French for future patient-interaction. However, this vignette demonstrates that it is much more than a mere language disconnect; there is a cultural disconnect that must be reconciled by understanding that people from other countries may not have the same educational experience, exposure to cultural/geographical icons, or life experiences. And it’s our role to be sensitive to that!
S18-40	Kanabur, “Breaking Down the Barrier” McDaniel, “Clinical Culture Shock: Low Health Literacy as a Barrier to Effective Communication”	[…] the ability for a physician to focus on the illness as well as the disease is invaluable in order to be a successful physician as well as to have a good relationship with the patient. The third essay, Clinical Culture Shock is also very important because it illuminates the critical nature of doctors seeing and relating to their patients as human beings, and not simply diseases to be cured.
S18-42	McDaniel, “Clinical Culture Shock: Low Health Literacy as a Barrier to Effective Communication”	This is one of the hardest truths to swallow as a medical student. We come into the field very excited in hopes of getting involved with unique medical interventions and grandiose ideas. Yet, some of the important and impactful work we will end up doing is simple comforting of patients or working with them and their families to guarantee care, comfort, and respect of patients ' wishes.
S18-45	McDaniel, “Clinical Culture Shock: Low Health Literacy as a Barrier to Effective Communication”	I do feel that within medicine today, there is some form of elitism, or at least disparities which, in accordance with socioeconomic barriers and disparities, put certain populations at a notable difference that has an adverse effect on their healthcare. As physicians, and purely as citizens, it is important to be cognizant of our privileges and disparities present in society and work towards bridging them.

### Theme 1: empathic conflict

In their essays, premedical students emphasized the theme of empathic conflict, focusing on the difficult balance between expressing emotion and empathy towards patients while maintaining an appropriate professional distance. Responses discussed how emotional expression enhanced the practice of medicine for both patients and physicians (F16-2, S17-1, S17-2, F17-40). Study participants reflected on the professional costs of empathic conflict and identified depersonalization, dehumanization, and emotional fatigue in the medical students’ blog posts (F16-1, S18-6, S18-7, S17-3). Students frequently reflected on the medical trainees’ challenge of balancing their emotional attachment to patients with a professionally appropriate empathetic response (F16-2), and expressed concern that achieving this balance would be challenging in their future careers (S18-6, F16-4, F16-15, S17-1, F17-31, S18-13). Several essays noted that emotional distancing was a defense mechanism (S18-2, S18-8) and that imposter syndrome was closely related to issues of empathic conflict and burnout (F17-7, F17-13). Students identified the importance of patient narratives in the practice of medicine (S17-4, S17-8, S17-10, S17-12, S17-22), and discussed the role these narratives may play in protecting the empathic wellbeing of medical students (F16-32, F17-19). Several essays also revealed how the near-peer blog posts exposed the students’ own internal empathic conflicts or conflicts they had personally witnessed in their interactions with other patients or clinicians, and how reflecting on those narratives allowed them to process their own emotions and empathy (S17-4, F16-1, F16-12, F16-17, F17-25, S18-38).

Jennifer’s internal struggle here is something that I’ve thought about a lot ever since

I decided to become a pre-med. At first, I used to wonder how doctors can maintain such a stoic outlook in the face of some clearly emotional decisions. What is the point of a brusque doctor? Shouldn’t we show some empathy and compassion to the patient who’s suffering? Why should treatment only be about the biological symptoms? I thought there was genuinely something wrong about doctors who failed to show empathy and compassion to their patients. It was detrimental. However, when I think about it now, I can kind of see why doctors may attempt to put some objectivity and emotional distance between themselves and the patient. Doctors are human beings. If you had to face illness and death regularly and if you were continually emotionally affected by it, doing your job as a physician would become very difficult. Keeping that emotional distance is not a weakness; it’s almost a necessity that allows a physician to work to the best of his or her ability. (F17-31)

### Theme 2: bias in healthcare

Premedical students’ writings demonstrated nuanced comprehension of the ways in which bias, whether overt, implicit, or structural, is present in medicine and reflected in their own personal experiences of and beliefs about race, discrimination, structural racism, and social justice (F16-21, F16-34, S17-23, F17-27, F17-35). Their essays commented on the *in-Training* bloggers’ descriptions of the subtle yet insidious ways that medical institutions discriminate against people of color, vulnerable populations, and marginalized cultures. The premedical students examined how their own implicit biases might influence the way that they would practice medicine in the future (F16-18, F16-30, F16-33, S17-13, S17-25, F17-11, F17-12, F17-32, S18-18, S18-29), and some advocated for coursework and training emphasizing the sociocultural dimensions of health and medicine (S17-14, F17-14, F17-20, S18-19). Others called attention to the need for institutional spaces and protocols better designed to serve diverse patient populations (F16-33, F16-41, S17-32, F17-33). Many students also took a broad, sociopolitical perspective on bias in healthcare, noting how systemic racism influences American medical practice, despite popular conceptions of medicine as an objective science (F16-24, F16-27, F16-33, F17-4, F17-20, S18-22, S18-29).

[T]he real issue isn’t the individuals who stick out for committing crimes of racism, but the system that made their implicit biases. This is a really important turning point in my own thoughts about this issue. ... [H]armful biases happen in everyday situations and though they aren’t as obvious, they matter and lead to statistically significant outcomes. (F17-4)

The fact that physicians constantly marginalize certain races (whether they are aware of it or not) when practicing motivates me to become more conscious of these acts when I go into the professional field. (S17-25)

### Theme 3: “the humanity of medicine”

Premedical students reflected on the medical student blog posts by discussing the complexity of the patient-physician relationship and expressing appreciation for what they, borrowing the phrase from *in-Training* blogger Jimmy Tam Huy Pham, refer to as the “human side of medicine” (2012). Their essays highlighted the professional importance of listening to the patient’s narrative and seeing patients as more than diseases to be cured (S18-32, S18-40). Premedical students reflected on their personal understanding of what makes a successful physician, focusing on the importance of open communication between doctors and patients, respecting patient’s wishes, understanding how patients live with medical conditions, and providing comfort and care to patients and their families (F16-33, F16-35, F17-21, S18-42). Several premedical students discussed aspects of medicine beyond the biomedical sciences that they recognized as critical to patient care, including social determinants of health (e.g. housing and health literacy), socioeconomic disparities, and barriers to accessing care (F16-49, S17-30, S18-29, S18-39). Some premedical students also used their essays to call for improvements in medical education, including increased training in addressing the inequalities that exist in both society and medicine (F16-18, F16-19, F16-31, S18-45, S18-29).

We come into the field very excited in hopes [of] getting involved with unique medical interventions and grandiose ideas. Yet, some of the most important and impactful work we will end up doing is simple[:] comforting of patients or working with them and their families to guarantee care, comfort, and respect of patients’ wishes. (S18-42)

When I volunteered in an emergency room, I witnessed the casual way in which patients who complained of pain were dismissed. I did not have access to medical records, but one patient I particularly remember was an older black lady who was crying because she was in pain. She said nobody had performed an examination on her, and when I asked a nurse, she waved me off, saying, “You get an eye for figuring out who’s faking it for drugs.” I was alarmed by this, but assumed she was correct, as I had almost no power in the situation. Whether or not this old woman was addicted to painkillers, wasn’t it the duty of medical professionals to trust her and give her the benefit of the doubt? When filtering through the hundreds of patients who are turned away for being fakers, isn’t it possible that a couple of them were telling the truth? (F16-33)

### Overarching theme: near-peer affinities

Our narrative analysis of the premedical students’ reflective essays also revealed unprompted expressions of near-peer affinities. Although students had not been instructed to reflect on the near-peer status of the *in-Training* authors, the essays in our dataset frequently linked the value of the blog posts to their status as narratives authored by near-peers. Representative quotations on near-peer affinities are displayed in Table 3. Students expressed feeling more optimistic and inspired about their future careers knowing that their near-peers were writing about empathy and humanism in medicine and stated that they felt they better understood the medical school experience after reading the near-peer blog posts (S18-14, F16-5, S17-10, S17-11). Premedical students often discussed their positive affinities towards near-peer works in contrast to works authored by faculty and written in more traditional scholarly form. Some noted that it was easier to connect with the narratives because they were authored by near-peers and praised the informal style of the blog pieces (F17-28, F16-5, S17-12, S17-4, F16-1, F16-7). Several authors appreciated the honesty and “raw” nature of the narratives (F16-7, F16-5, F16-1, F16-11). However, there were also criticisms of the near-peer pieces, including that they lacked literary technique and style (F16-1, F16-6, F16-9, F16-8) and were not sufficiently nuanced in their discussion of the core humanities issues (F16-23, F16-31).

The fact that I felt emotionally involved […] is a testament to the value of narratives. Narratives from medical students are particularly relevant to us pre-meds, because we can easily imagine ourselves in their shoes a few years in the future. (S17-4)

While reading the selections from *in-Training* my initial reaction was surprise at their raw nature. Although each short story or poem had a clear central argument transmitted through the emotional perspective of the medical student author, they lacked the literary style and techniques I have come to expect. At first, I felt disappointed by this fact, feeling that the lack of polish took away from the worth of the selections. However, after just a couple narratives, I came to appreciate how intimate this informal style of writing can be. Since the stories are not excessively formulated or contrived, thoughts and emotions behind their writing are allowed to shine through. (F16-1)

**Table 3:** Selected quotations from pre-medical student essays on near-peer affinities

Essay Identifier	Reflective Essay Excerpt
F16-1	While reading the selections from *in-Training* my initial reaction was surprise at their raw nature. Although each short story or poem had a clear central argument transmitted through the emotional perspective of the medical student author, they lacked the literary style and techniques I have come to expect. At first, I felt disappointed by this fact, feeling that the lack of polish took away from the worth of the selections. However, after just a couple narratives, I came to appreciate how intimate this informal style of writing can be. Since the stories are not excessively formulated or contrived, thoughts and emotions behind their writing are allowed to shine through.
F16-5	The honesty from these medical students really stands out throughout these narratives […] These medical students reveal the concerns and challenges that I have always suspected would be hardest for me; however, I am comforted by the fact that these students use their experiences to grow and become stronger and better doctors.
F16-6	I found the stories to be interesting and touching, but it was an interesting adjustment to reading fiction that was not crafted by a seasoned creative writer. Some of the writing still felt very clinical or over-flourished with language that didn’t fit the situation in the story. For example, I thought the story “Exam Room 3” told a very emotional and thought-provoking story, but the language felt forced, especially in phrases like, “my brain moved like molasses,” and “the anguished sobs stopped me in my tracks.” This is not to say that any of the writing styles in these selections negated their relevance in the field of the medical humanities. I think that understanding the experience of practicing medicine is just as much a part of studying the medical humanities as is understanding the experience of the patient. I value the stories given in this reading for their unique first-person perspective, but I don’t think I’d spend time close reading the language in these narratives.
F16-7	I enjoy reading these *in-Training* selections, because of the rawness and honesty of the writers.
F16-8	Overall, I was relatively impressed with the quality of the writing in these essays, though I did not find them to be of the same quality as those written by more experienced physician-writers like Richard Selzer.
F16-9	I would be interested to see what kind of workshopping goes into these stories after their initial writing. In previous discussions, I’ve learned of writing groups or special guided editing workshops that can help authors engage with the emotions involved with their writing process and tailor their writing to showcase that. I was intrigued by the amount of medical jargon included in the text, especially when I compare it to the works that we read in class on Thursday as a part of our guest lecture. Those works appeared to be more stripped down and invested in emotion and metaphor. I do think that these works have some instructional value, but I wonder if a more engaged writing process could have yielded more cohesive work. I found that I could feel the themes of the story itself, but it was hard to connect with the writing style, which often felt cold and flat. It seemed that the authors understood which stories were particularly impactful, but they did not channel them effectively in my opinion.
F16-11	The emotion is very raw because for medical students, experiences are still new. Nothing is mundane yet.
F16-23	I know it’s difficult to tackle such an expansive topic in a single paper, but I still found some ideas to be only vaguely addressed and devoid of nuance.
F16-31	A fault I see in Jennifer Tsai’s piece, however, is that she puts a significant amount of emphasis on systemic biases in the hospital setting, an emphasis that I think is overestimated. Physicians are also at least partly culpable for the biases they inflict on their patients, and explaining the problem away by using the idea of systemic racism does not solve the issue. Instead, medical schools must make a greater effort to educate their students in understanding the patient as a whole, including their sociopolitical environment and their cultural norms, so that physicians in the real world are equipped to deal with a diverse patient population and can fight structural biases.
F17-28	This was my favorite reading assignment so far. It was very easy to connect with the medical student and feel whatever internal conflict or emotions they struggled through. I thought it was especially helpful because I will likely never be in any of these exact situations and definitely don’t have the exact same background or perspective as any of them, so it allows me to understand the lessons they’ve learned without having to actually live through all those experiences myself. I especially liked the emphasis on patient connection and empathy throughout the stories. I felt it shed a positive light on caring about a patient.
S17-4	The fact that I felt emotionally involved however, is testament to the value of narratives. Narratives from medical students are particularly relevant to us pre-meds, because we can easily imagine ourselves in their shoes a few years in the future. The naivety of these stories sometimes struck me – many pre-meds aspire to save lives and cure illnesses, and yet the reality often strays far from that ideal.
S17-10	All of the narratives in this book are incredibly detailed, poignant. I was moved by each and every one of them and now feel like I understand a part of medical school/doctoring that I hadn't considered would be so impactful before.
S17-11	Put simply, each medical student’s experience thus far was different despite some common themes and underlying ideas. As such, the book as a whole presents a balanced idea of what it is like to be on the brink of starting your career, to be overwhelmed with newness and a sense of purpose, and to be at the bottom of the medical totem pole. I loved this book. Each reading spoke to me in a different but moving and special way. The stories were rich and diverse in themes, stories, and concepts, ranging from the idea of humor in the patient experience in “the Chair” by Ben Ferguson to the meaning of death for someone whose life is already compromised as in “Fading Memories of Love and Martinis” by Joshua Niforatos. Each author was candid, thoughtful, and eloquent in voicing their good days and bad days at the start of their medical career. As an aspiring physician myself, I found this inspiring. I hope to write and never stop writing in my own career one day. It not only gives you perspective and understanding of your purpose, your passion, and your experiences but also provides the rest of the world insight on the doctor side of the patient experience. I already said this but I’ll say it again: I loved this reading and even read many other stories in the book. It is so generous of these doctors to share these ideas with us. What a privilege to get to read these.
S17-12	These series of stories in *in-Training* is the most interesting reading we had to do for this class so far because these are more personable, approachable, and recount real life experiences. Because I want to be a doctor as well, it gives me a myriad of unique perspectives that can prepare me for what to expect in the future. It ties in very nicely to the topics we are discussing in class and the other class textbooks, but the stories are just more relatable. I feel like I understand topics like death and doctor-patient relationships better from reading these stories.
S18-14	If these readings provided me with any consolation, it is knowing that all of these thoughtful, empathic anecdotes were written by current or recent medical students. These students were aware of this need to add humanity back into the equation of medicine, whether this be through empathizing with the patient’s history or improving their patient bedside etiquette. They acknowledge their limits as physicians, not being able to resolve their patients’ personal issues or illnesses that are beyond their control, but that doesn’t deter them from trying to provide the best form of care that they can.

## Discussion

Our analysis found the student responses to the near-peer medical student narratives from *in-Training* to be significant in two ways. First, the core themes identified in the premedical student essays demonstrate engagement with landmark topics in the humanities education of medical trainees: empathic conflict, bias in healthcare, and the humanity of medicine. Second, the students frequently discussed the medical student blog posts through the lens of self-reflection, closely identifying with the near-peer bloggers’ experiences and considering how they would respond to the same situations. While the first finding confirms our hypothesis regarding the utility of near-peer narratives for introducing core concepts in the medical humanities and fostering self-reflection on the professional demands of a career in medicine, the second was an unexpected finding that further confirms the pedagogical value of integrating near-peer blogs in an undergraduate premedical humanities course. Both findings highlight the importance of writing personal narratives as medical students and reading those narratives as premedical students.

Our analysis of the “near-peer affinities” present in undergraduate premedical students’ reflective writing suggests that near-peer narratives expose premedical students to varied perspectives, which serves to strengthen and broaden their personal experiences from clinical shadowing or volunteering. The integration of near-peer narrative may play a significant role in cultivating premedical students’ professional identities by attending to the demands of the profession and the social contexts in which medicine is practiced. Through their reflections on near-peer writings, these students enter medical school having developed an appreciation for the humanistic aspects of medicine and an ability to reflect on the role and duties of the physician in society. Structured engagement with near-peer writings in the undergraduate medical humanities curriculum, our research suggests, can be particularly influential in premedical students’ professional identity development because it provides those who are contemplating a career in medicine the opportunity to learn from the collective experiences of medical trainees.

For educators, providing curricular material that is more easily relatable to undergraduates presents an obvious advantage in maximizing student engagement. However, this should be balanced with recognition of the novice nature of many near-peer pieces, as several study participants mentioned that the quality of the writing was at times inferior to the other reading assignments in the curriculum that were written by more experienced authors. At the same time, by demonstrating their ability to critique the quality and purpose of the medical student posts, premedical students in our study demonstrated their willingness and ability to engage in key discourses within the medical humanities.

### Theme 1: empathic conflict

Our finding that many study participants wrote self-reflective essays on the topic of empathic conflict is an important outcome of this study and reveals that premedical students can discuss challenges with empathy and emotional distancing through the lens of narrative medicine in the same way as medical students. By engaging with near-peer blog posts, premedical students recognized their vulnerability to empathic conflict and the challenges with emotional distancing, and how this conflict may affect their connections with future patients and with their own personal wellbeing, issues that are well-represented in the literature on this subject (Hirsch 2007; Eikeland et al. 2014; Andersen et al. 2020). Students also recognized that reflecting on the narratives themselves was cathartic and instrumental in their own professional development. Our analysis further revealed that study participants discussed both the positive and negative effects of medical students’ empathic engagement with patients and their stories and included nuanced perspectives on the structural causes of burnout, including depersonalization and imposter syndrome. Self-reflective writing normalizes discussion of empathic conflicts, which has been shown to be critical for building resilience and combating burnout in health professionals (Chen and Forbes 2014; Narayan, Stern, and Fornari 2018; Harscher et al. 2018; Pohontsch et al. 2018). Our finding that premedical students were able to recognize burnout among their near-peers, despite not experiencing burnout themselves, may have important educational implications in allowing them to better recognize and prevent burnout in themselves in the future (Jennings 2009).

### Theme 2: bias in healthcare

The social construction of race, racial health disparities, and the persistent legacy of racism in United States health systems are frequently explored topics within health humanities research, and efforts toward racial justice within health professions education is rooted in the health humanities: ethics, medical sociology, and the history of medicine (Nieblas-Bedolla et al. 2020). Students’ essays exploring racial bias in medicine, socioeconomic disparities limiting access to healthcare, and the linguistic or cultural barriers that impact patient-provider communication provide compelling evidence that premedical students’ reflections on near-peer writing can effectively encourage a deeper awareness of these topics and the development of cultural competence as defined by the AAMC. The students in our study exhibited a significant willingness to engage with near-peer writings on these complex issues rather than avoiding recognition of medicine’s imperfections, evidence of their growing “structural competence” on issues of systemic racism, per Petty and colleagues (2017). In their essays, students further reflected on how near-peer narratives resonated with their own or their loved ones’ experiences with bias in a healthcare setting and considered how their own implicit biases might impact their ability to provide care for their patients. We find, therefore, that reflecting on near-peer narratives provides a productive space for undergraduate premedical students’ personal and professional development even before entering formal medical education, especially as it pertains to implicit bias and structural racism.

### Theme 3: the “humanity of medicine”

Our analysis finds that near-peer narratives are an effective means of encouraging students to relate to their patients as people, and not just as constellations of symptoms to be cured. Entering medical students are asked to appreciate that an individual’s health and well-being are determined by the intersection of biological, social, cultural, and psychological factors. Premedical students often shadow physicians, volunteer in clinical settings, or work in ancillary services as emergency medical technicians or scribes to develop this understanding (Association of American Medical Colleges, “Five Ways to Gain Experience without Shadowing”). Our findings show that near-peer written works can serve as an additional avenue for premedical students to reflect on their personal experiences with patients in a nascent form of narrative medicine. Students described the complex social and cultural narratives of the patients whom they had met in their premedical experiences, and how reading the near-peer pieces taught them to reflect more critically on their encounters with patients and on the structural issues within medicine and health care that contributed to these illness narratives. The premedical students in our study also reflected on how they personally would approach the patient-physician interactions and challenges described by their medical student near-peers, which is a key critical thinking skill required of medical professionals. Through their reflections, premedical students contemplated the role and duties of the physician in today’s society, and how they will approach the intersection of biomedical sciences and the “human side of medicine” as they enter the medical field.

### Overarching theme: near-peer affinities

Although students were not explicitly instructed to comment on the authorship of the *in-Training* narratives, our analysis revealed frequent reflection on the authors’ status as near-peers. These commentaries included both positive responses to reading near-peer works as well as discussions that were more critical of the quality of novice writers. Near-peer narratives are, by the students’ own estimation, accessible, engaging, and impactful introductions to foundational topics in the medical humanities. Student reflections address the pleasure of near-peer learning through narratives that were written by trainees close in academic level and age to undergraduate premedical students. Thus, we find that near-peer narratives published in online, open-access venues (e.g. blogs) serve as a crucial introduction and complement to more traditional academic prose published in peer-reviewed venues (e.g. scholarly journals, monographs, anthologies, or edited collections) for premedical students.

### Limitations

This study has several limitations. Research was conducted in only one institution, and while the study population was racially and socioeconomically diverse, it may not be statistically representative of all premedical students across United States universities and colleges. As a qualitative analysis of an educational intervention, the study method did not include randomization or a comparator group, raising the possibility of selection bias among study participants. The students who participated in the study were enrolled in a medical humanities course and are not necessarily representative of the general population of premedical students. Finally, readings from *in-Training* were chosen for their value in premedical health humanities education and therefore are not representative of all possible near-peer creative nonfiction narratives or blogs, which may not have equal value in achieving the results described in this study.

## Conclusions

This study identifies a novel pedagogical method for introducing core topics in the medical humanities through the integration of an online near-peer narrative medicine blog to augment traditional medical humanities teachings for premedical students. The premedical student essays analyzed in our study demonstrated nuanced engagement with three core themes – empathic conflict, bias in medicine, and the human side of medicine – as well as strong affinities with near-peer authors. Our results demonstrate how reading and writing about near-peer narrative accounts of medical training can provide a unique and valuable perspective on the impact of patient encounters, particularly those involving vulnerable and marginalized populations, as they shape professional identity formation for premedical and medical students. Our qualitative assessment of student responses to the *in-Training* readings thus underscores the value of this pedagogical method. This study finds that near-peer learning through engagement with creative nonfiction published on a medical student blog allows for critical reflection on medical education from the profession’s future leaders. The ability to use near-peer narratives to facilitate empathic learning, facilitate resilience, and address bias in medicine is an opportunity that warrants further study and integration into existing premedical educational curricula.

## Declarations

### Endnote

^1^ This phrase is drawn from the final line of Jimmy Tam Huy Pham’s essay, “A Night at the Homeless Shelter,” which was quoted frequently in students’ reflective essays.
